# Lactic Acid Bacteria Exopolysaccharides Unveiling Multifaceted Insights from Structure to Application in Foods and Health Promotion

**DOI:** 10.3390/foods14050823

**Published:** 2025-02-27

**Authors:** Wei Liu, Yajun Wei, Rong Xiang, Bo Dong, Xi Yang

**Affiliations:** 1 Zhejiang Provincial Key Laboratory of Agricultural Microbiomics, Institute of Plant Protection and Microbiology, Zhejiang Academy of Agricultural Sciences, Hangzhou 310021, China; biolwei@sina.com; 2 Institute of Animal Science, Guangdong Academy of Agricultural Sciences, Guangzhou 510640, China; weism4224@163.com; 3 Institute of Animal Health, Guangdong Academy of Agricultural Sciences, Guangzhou 510640, China; xiangrong@gdaas.com; 4 State Key Laboratory of Swine and Poultry Breeding Industry, Agro-Biological Gene Research Center, Guangdong Academy of Agricultural Sciences, Guangzhou 410640, China; dongbo@gdass.com

**Keywords:** lactic acid bacteria, exopolysaccharides, biological functions, food applications, health benefits, biosynthesis

## Abstract

Lactic acid bacteria (LAB) exopolysaccharides (EPSs) have garnered significant scientific interest due to their multifaceted roles in food technology and health promotion. This comprehensive review systematically examines the structural classification of LAB EPSs, emphasizing distinctions between homo-and heteropolysaccharides, as well as the influence of substituent groups (e. g., acetyl, phosphate) on their physicochemical and bioactive properties. Advanced isolation methodologies, including ethanol precipitation and ultrafiltration, coupled with characterization techniques such as nuclear magnetic resonance (NMR) spectroscopy and atomic force microscopy (AFM), are vital for deciphering their chemical and physical characteristics. The biosynthesis pathway, governed by *eps* operons and modulated by environmental factors (e.g., carbon sources, Ca^2+^), are discussed as targets for genetic engineering to enhance yield and functionality. Functionally, LAB EPSs display antioxidant, immunomodulatory, anti-tumor, anti-viral, and anti-biofilm activities, with demonstrated applications as natural additives in the food industry, prebiotics, and drug delivery systems. Despite their potential, challenges such as cost-effective production and regulatory hurdles persist. Future research should prioritize the elucidation of molecular mechanisms, clinical validation of health claims, and sustainable bioprocessing innovations to fully harness the transformative potential of LAB EPSs across food, pharmaceutical, and agricultural industries.

## 1. Introduction

Lactic acid bacteria (LAB) have been utilized in food fermentation processes for centuries, with their beneficial effects on food quality and preservation well-documented [1]. However, the study of exopolysaccharides (EPSs) produced by LAB represents a relatively more recent and rapidly evolving field of research. The initial investigations into LAB EPSs date back to the mid-20th century when their presence was first identified in fermented dairy products [1]. Since then, research on LAB EPSs has steadily expanded.

The biological functions of LAB EPSs are diverse and of great significance. In food matrices, they contribute to the texture, viscosity, and stability of products. For example, in fermented dairy and bakery items, EPSs can enhance mouthfeel and shelf life [2]. Beyond food, LAB EPSs have shown potential in modulating the gut microbiota, thereby influencing host health. They can act as prebiotics, promoting the growth of beneficial gut bacteria and potentially affecting immune function and metabolism [3,4]. Recent studies have also demonstrated that certain LAB EPSs can interact with immune cells directly, modulating cytokine production and enhancing immune responses [5,6].

In terms of application, LAB EPSs are widely used in the food industry as natural additives. They are incorporated into various products like yogurts, cheeses, and beverages to improve their organoleptic properties [7]. Moreover, emerging research is exploring their use in pharmaceuticals and biomedical fields. For instance, they could serve as carriers for drug delivery systems due to their biocompatibility and biodegradability [8]. Additionally, a new study has reported the potential of LAB EPSs in promoting skin wound healing by enhancing cell migration and collagen synthesis [9].

The mechanism research of LAB EPSs has also evolved over time. Early studies focused on basic isolation and characterization techniques. Recently, advanced molecular and omics technologies have been employed to elucidate the biosynthesis pathways and the precise interactions between EPSs and host cells or the gut microbiota. These latest advancements hold great promise for a more comprehensive understanding and exploitation of LAB EPSs in the future.

## 2. Classification of EPSs from LAB

The classification of EPSs from LAB is based on multiple aspects including structure, type, and substituent groups, which contribute to their diverse properties and functions (Table 1).

### 2.1. Structure-Based Classification

**Figure 1:**
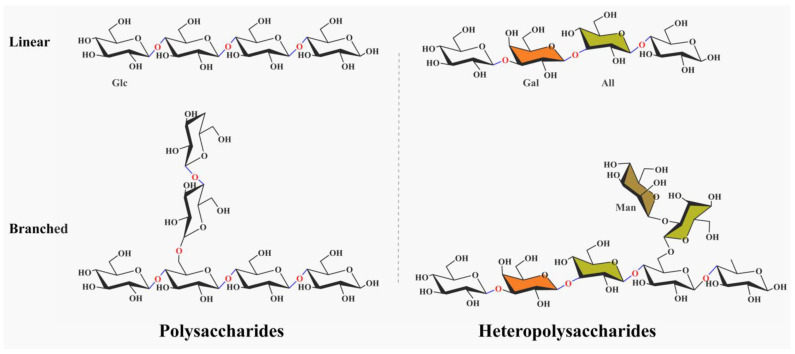
Structural classification of LAB exopolysaccharides (EPS). Linear EPS (e.g., dextran, β-1,4-linked glucose) and branched EPS (e.g., *Lactobacillus plantarum* EPS with β-1,6 side chains) are depicted. Homopolysaccharides (single sugar type) and heteropolysaccharides (multiple sugars) are differentiated by color: white/transparent (glucose), orange (allose (All)), green (galactose (Gal)), brown (mannose (Man)).

EPS structures can be either linear or branched, as demonstrated by studies on dextran (linear) and *Lactobacillus plantarum* EPS (branched) [14,17]. Linear EPSs, such as glucans, exhibit unbranched chains of sugar monomers linked by β-1,6 or β-1,4 glycosidic bonds [12], while branched EPSs feature complex side-chain architectures that influence solubility and rheological properties [12,17] (Figure 1). Some dextran molecules have a linear structure composed mainly of glucose units linked in a specific pattern. Branched EPSs, on the other hand, possess side chains attached to the main sugar chain. This branching significantly affects their physical and chemical properties. Branched EPSs often have a more complex three-dimensional conformation, which can influence their solubility, viscosity, and interaction with other molecules. The degree of branching and the length of the branches can vary among different LAB strains, leading to a wide range of rheological behaviors in food and biological systems [12,17].

### 2.2. Type Classification

LAB EPS are mainly divided into homo-polysaccharides and hetero-polysaccharides [11]. Homo-polysaccharides, such as dextran and levan, consist of a single type of sugar monomer. As mentioned, dextran is composed solely of glucose units. It has good water solubility and can form gels under certain conditions, which is beneficial for applications in food texture modification. Levan is composed solely of fructose polymers, and it shows potential as a prebiotic due to its fermentability by beneficial gut microbiota [11,13]. These polymers are synthesized via repetitive glycosidic linkages (e.g., β-1,6 in dextran) and are widely used in food and biomedical applications due to their predictable physicochemical properties [13,15]. Hetero-polysaccharides are composed of multiple sugar monomers. For instance, some hetero-polysaccharides contain glucose, galactose, and mannose. They usually have more intricate structures and can exhibit unique biological activities. A particular hetero-polysaccharide may have immunomodulatory properties, which are related to its specific sugar composition and linkage patterns [13].

### 2.3. Substituent Group Classification

The presence and nature of substituent groups, such as acetyl or phosphate moieties, critically influence EPS functionality. Some EPSs may have acetyl groups attached to their sugar monomers. These acetyl groups can affect the hydrophobicity and charge of the EPS molecule. For example, EPSs with acetyl groups from *Streptococcus thermophilus* exhibit enhanced hydrophobicity and metal-binding capacity, while the phosphate groups on EPSs from *Lactobacillus plantarum* demonstrate improved immunomodulatory activity [10,15,16].

In summary, the classification of LAB EPSs based on structure, type, and substituent groups is essential for understanding their properties and potential applications. Different combinations of these characteristics result in a vast array of EPSs with unique functions in food and health promotion.

## 3. Isolation and Characterization of EPSs

**Figure 2:**
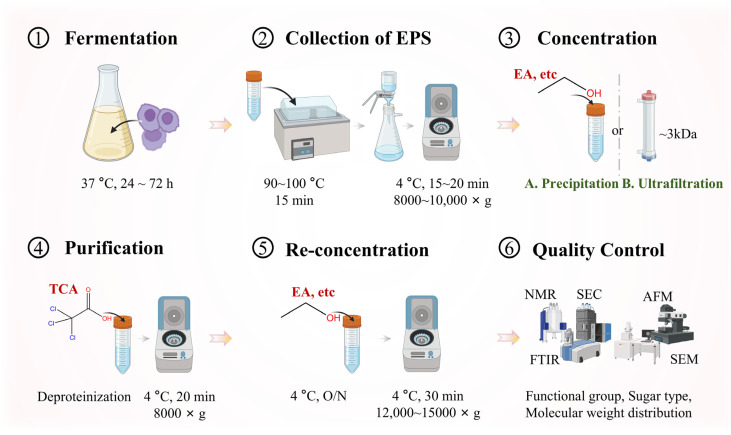
Schematic workflow for the isolation and purification of LAB exopolysaccharides (EPSs). The process begins with strain screening using mucoid colony selection on agar media or colorimetric assays to identify high-yield EPS-producing LAB strains. Following fermentation, cells are removed via centrifugation, and EPSs are precipitated from the supernatant using ethanol or isolated via ultrafiltration. Subsequent characterization employs Fourier-transform infrared spectroscopy (FTIR) for functional group analysis, nuclear magnetic resonance (NMR) for structural elucidation (monosaccharide composition, glycosidic linkages, and substituents), and size exclusion chromatography (SEC) for molecular weight profiling. Scanning electron microscopy (SEM) and atomic force microscopy (AFM) provide morphological insights, resolving surface topology and aggregation states. This integrated approach ensures comprehensive physicochemical and functional evaluation of LAB EPSs.

LAB exopolysaccharide isolation and characterization is important for understanding EPSs’ nature and potential. Screening methods, such as mucoid colony selection on agar media or colorimetric assays for extracellular sugars, are employed to identify high-yield strains [1,18]. Subsequent isolation techniques, including ethanol precipitation and ultrafiltration, ensure purity for downstream analyses [18,19]. It commences with the screening of LAB strains for EPS production, followed by isolation methods to obtain pure EPS samples. Subsequently, various characterization techniques are employed to comprehensively analyze the chemical and physical properties of the isolated EPSs, providing essential insights for further research and applications (Figure 2).

### 3.1. Screening

The screening of LAB strains for EPS production is the initial step. This can be achieved through various methods [1,18]. One common approach is to culture a large number of LAB isolates under specific growth conditions and then observe the formation of mucoid colonies, which is an indication of EPS production. For example, in a study by Li et al. [1], a collection of LAB strains was plated on agar media supplemented with specific sugars. After incubation, colonies with a slimy or mucoid appearance were selected for further analysis. Another screening method involves using colorimetric assays to detect the presence of sugars in the culture supernatant, which may be indicative of EPS secretion.

### 3.2. Isolation

Once the EPS-producing strains are identified, the isolation process begins with three sequential steps:

#### 3.2.1. Centrifugation

A typical isolation method involves culturing the selected LAB strain in a liquid medium. After fermentation, the cells are removed from the culture via centrifugation at a suitable speed, usually around 8000–10,000× *g* for 15–20 min, a method validated in [19,20]. The resulting supernatant containing crude EPSs is retained for further processing.

#### 3.2.2. Ethanol Precipitation

Ethanol precipitation remains the gold standard for polysaccharide isolation. The supernatant is slowly added to a cold ethanol solution (usually 2–3 volumes of ethanol) under gentle stirring. The mixture is then left at 4 °C overnight to allow the EPSs to precipitate [20]. The precipitated EPSs are collected via centrifugation at a higher speed, around 12,000–15,000× *g*, for 30 min. This method has been successfully implemented in recent EPS isolation from yogurt-derived LAB strains, yielding substantial quantities of purified EPSs [19,20].

#### 3.2.3. Ultrafiltration

As an alternative approach, ultrafiltration employs a membrane with a specific molecular weight cut-off (typically 10–100 kDa) to retain high-molecular-weight EPSs while eliminating low-molecular-weight contaminants [18]. The EPSs, which have a relatively large molecular weight, are retained on the membrane, while smaller molecules and impurities pass through.

### 3.3. Characterization

Characterization of the isolated EPSs is crucial to understand their properties. There are several approaches to decode structural and functional attributes (Table 2). Fourier-transform infrared spectroscopy (FTIR) rapidly identifies the functional groups (e.g., hydoxyl, carbonyl) present in the EPSs, but it lacks linkage specificity, necessitating complementary techniques like nuclear magnetic resonance (NMR) for detailed monosaccharide sequencing [17,21]. Scanning electron microscopy (SEM) and atomic force microscopy (AFM) further resolve morphological features, such as surface roughness and aggregation states [22,23]. Nuclear magnetic resonance (NMR) spectroscopy provides detailed information about the sugar composition and the linkage patterns between the sugar monomers. NMR was used to determine that a specific EPS was composed of glucose, galactose, and mannose in a particular ratio and with specific alpha and beta linkages [17,21]. Size exclusion chromatography (SEC) is employed to determine the molecular weight distribution of the EPSs. By comparing the elution time of the EPS sample with that of standard polymers of known molecular weights, the average molecular weight and the polydispersity index of the EPSs can be calculated. Additionally, scanning electron microscopy (SEM) and atomic force microscopy (AFM) can be used to visualize the morphology and surface characteristics of the EPSs. SEM images can show the overall shape and aggregation state of the EPSs, while AFM can provide high-resolution images of the surface topography, revealing details such as the presence of pores or irregularities. Generally, FTIR is preferred for rapid functional group screening, whereas NMR provides unparalleled structural detail but requires pure samples [17,21]. For molecular weight profiling, SEC offers high throughput but must be paired with complementary techniques like light scattering to avoid biases from aggregation [18]. SEM and AFM are complementary for morphological studies: SEM captures bulk surface features, while AFM resolves nanoscale topography and mechanical properties [23,24] (Table 2).

## 4. EPS Biosynthesis

### 4.1. Molecular Pathway and Regulatory Elements

**Figure 3:**
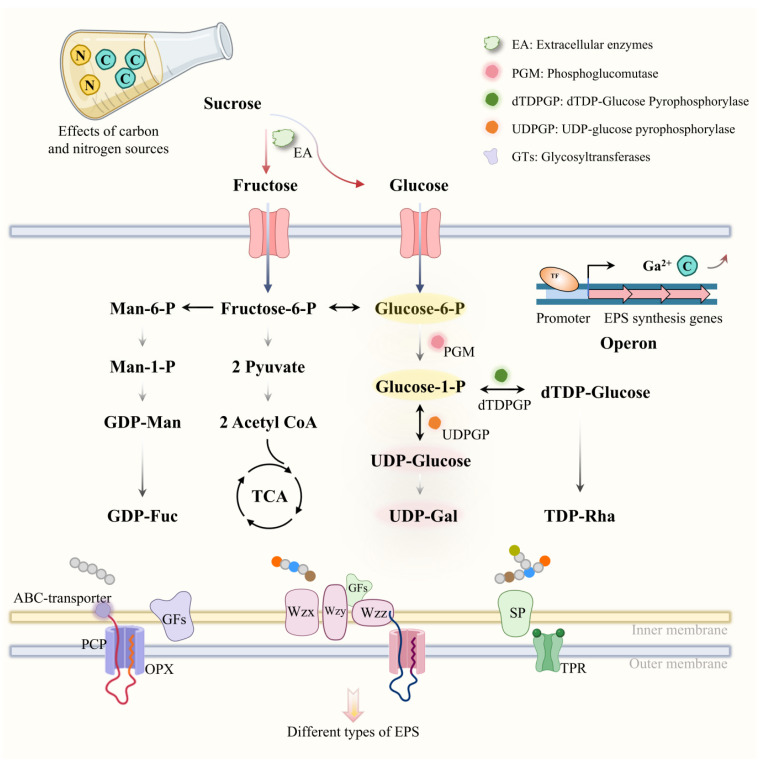
Molecular pathway and regulatory network governing LAB EPS biosynthesis. EPS synthesis initiates with the activation of sugar nucleotides (e.g., UDP-glucose), which serve as substrates for glycosyltransferases (GTFs). These enzymes catalyze polymerization, forming linear or branched polysaccharide chains. The *eps* operon (e.g., *epsA–E* and *epsR*) orchestrates biosynthesis: *epsA* encodes a priming GTF for chain initiation, while *epsB–E* mediate polymerization and export. Environmental factors, such as carbon source availability and Ca^2+^ concentration, modulate transcriptional regulation via epsR, enhancing GTF stability and altering monosaccharide ratios. Calcium ions (20 mg/L) further optimize enzyme–substrate interactions through calcium binding domains in GTFs. Genetic engineering strategies (e.g., promoter optimization) are highlighted to improve yield and functionality.

The biosynthesis of EPSs in lactic acid bacteria (LAB) involves a series of enzymatic reactions and genetic regulatory mechanisms. The initial step includes the activation of sugar nucleotides (e.g., UDP-glucose), which serve as the building blocks for the polysaccharide chain assembly. Glycosyltransferases (GTFs) catalyze the polymerization of these monomers, determining EPS structure and type [25] (Figure 3).

Recent studies have highlighted the role of specific genetic clusters in EPS biosynthesis. For instance, In *Lactococcus lactis*, the *eps* operon (e.g., *epsA-E* and *epsR*) plays a central role in EPS biosynthesis. The *epsA* gene encodes a priming glycosyltransferase that initiates chain elongation, while *epsB–E* are responsible for polymerization and export [27,28]. The transcriptional regulator epsR responds to environmental signals such as carbon availability and pH, modulating GTF expression and EPS branching patterns [27]. Additionally, specific carbon sources mediated by a transcriptional activator unregulated EPS biosynthesis in LAB strains. Calcium ions (Ca^2+^) at 20 mg/L were shown to enhance EPS yield in *Lactobacillus plantarum* by stabilizing GTFs and altering monosaccharide composition (e.g., increasing glucose/galactose ratios) [16]. This Ca^2+^-dependent regulation is mediated through specific calcium binding domains in GTFs, which optimize enzyme–substrate interactions [16]. Such findings highlight opportunities for genetic engineering (e.g., promoter optimization or *epsR* overexpression) to enhance EPS production.

### 4.2. Fermentation

Fermentation conditions are pivotal in determining the yield, structure, and functional properties of LAB-derived exopolysaccharides (EPSs). Batch fermentation remains the most commonly used method. Optimization of parameters such as carbon sources, nitrogen, PH, temperature, agitation, and fermentation duration is essential for maximizing EPS production. Below, we expand on these factors with recent insights and integrate findings from reference [29], which employs response surface methodology (RSM) to analyze the interplay of critical variables (Table 3).

The carbon source is a crucial factor that impacts EPS yield due to its role as a precursor for sugar nucleotide biosynthesis. Glucose is a widely used carbon source, but other sugars such as fructose and sucrose can also be utilized. For example, *Lactobacillus casei* produced 12.14 g/L EPS using glycerol as the carbon source, significantly higher than yields with glucose (8.79 g/L) or molasses (6.25 g/L) [30,31]. Similarly, the nitrogen source also affects EPS synthesis by regulating enzyme activity. Organic nitrogen sources like peptone and yeast extract are preferred over inorganic alternatives, as they provide essential amino acids and cofactors for glycosyltransferases (GTFs) [32].

Temperature and pH modulate both microbial growth and enzymatic activity. LAB generally grow well in a temperature range of 25–40 °C. However, the optimal temperature for EPS synthesis may vary among different strains. *Lactobacillus paracasei* exhibits maximal EPS production at 30 °C, where GTSs and membrane-bound export proteins remain stable [32]. Most LAB prefer a slightly acidic pH range of 5–7. A change in pH can alter the conformation and activity of the glycosyltransferases and other enzymes, thereby influencing EPS production [33]. Reference [29] corroborates these findings, demonstrating that Propionibacterium acidi-propionici achieves peak EPS synthesis at pH 6.5 and 30 °C.

In addition to these factors, agitation ensures uniform distribution and prevents cell clumping, particularly in high-viscosity EPS-containing cultures. For example, *Streptococcus thermophilus* requires 100–200 rpm agitation to maintain homogeneous mixing, achieving 9.20 g/L EPS under these conditions [33]. However, excessive agitation may shear EPS chains, reducing molecular weight and functional properties. LAB are typically facultative anaerobes, and while microaerobic conditions can enhance biomass growth, strict anaerobic environments are often optimal for EPS synthesis to minimize oxidative stress on GTFs [32].

EPS production is growth phase-dependent, with most LAB strains synthesizing EPSs during the late exponential or stationary phase. Prolonged fermentation (>48 h) may lead to enzymatic degradation of EPSs or nutrient depletion, reducing yield. For instance, *Lactobacillus plantarum* achieves maximal EPS accumulation at 36 h, beyond which yields plateau or decline due to autolytic enzyme activity [16].

Advanced statistical tools like response surface methodology (RSM) have been employed to optimize fermentation parameters. In reference [29], RSM was used to model the combined effects of temperature (25–35 °C), pH (5.5–7.5), and yeast extract concentration (5–25 g/L) on EPS production by *Propionibacterium acidi-propionici*. The study identified interactive effects, such as the synergistic enhancement of EPS yield at moderate temperatures (30 °C) and pH 6.5, providing a framework for multi-variable optimization in industrial settings.

**Table 3:** Comparative analysis of fermentation conditions for LAB EPS production.

LAB Strain	Carbon Source	Temperature (°C)	pH	Agitation (rpm)	EPS Yield (g/L)	Key Mechanism	Reference
*Lactobacillus casei*	Glycerol	37	6.5	150	12.14	Glycerol enhances sugar nucleotide synthesis	[31]
*Lactobacillus paracasei*	Glucose	30	6.0	200	8.79	Mild acidity stabilizes GTFs	[32]
*Streptococcus thermophilus*	Sucrose	40	5.5	100	9.20	Thermotolerant GTFs enable high-temperature synthesis	[33]

## 5. Biological Function

### 5.1. Antioxidant Function

**Figure 4:**
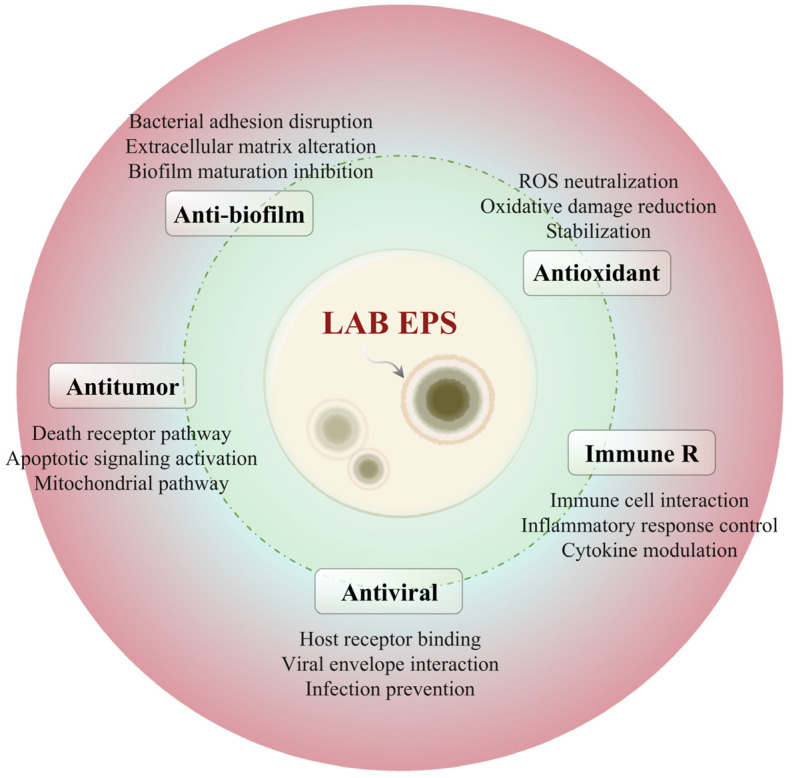
Biological functions of LAB EPSs, including antioxidant, immunomodulatory, and anti-biofilm activities. ROS: reactive oxygen species, Immune R: immune response, Anti-biofilm: anti-biofilm activity.

LAB EPSs exhibit potent antioxidant properties (Figure 4), scavenging free radicals (e.g., superoxide anions, hydroxyl radicals) via redox-active hydroxyl and carboxyl groups [34,35]. In vivo studies demonstrate their efficacy in reducing lipid peroxidation in murine liver tissues, underscoring their potential to mitigate oxidative stress-related pathologies [35,36]. The antioxidant activity of EPSs is attributed to the presence of hydroxyl and other functional groups in their chemical structures. For example, one study [34] demonstrated that EPSs isolated from a specific strain of LAB were able to reduce the levels of superoxide anion radicals and hydroxyl radicals in a cell-free system. In vivo experiments using animal models have also provided evidence of the antioxidant effects of LAB EPSs. In studies on mice fed with LAB EPSs, the levels of lipid peroxidation in their liver tissues were significantly decreased compared to the control group, indicating the protection of cells from oxidative damage [35,36]. Recent comparative analyses have revealed that polysaccharides from plant sources (e.g., pumpkin and Angelica sinensis) share similar redox-active moieties, suggesting conserved structure–activity relationships across biological kingdoms [1,37,38]. This antioxidant function of LAB EPSs is of great importance, as oxidative stress is associated with numerous diseases, including cardiovascular diseases and neurodegenerative disorders.

### 5.2. Immunity Regulation

One of the well-studied biological functions of LAB EPSs is their immunomodulatory effect. They can interact with various components of the immune system. EPSs can enhance the phagocytic activity of macrophages. For instance, research on a particular LAB EPS showed that it increased the engulfment of pathogens by macrophages in vitro [39,40,41,42] Additionally, LAB EPSs can modulate the production of cytokines. They can stimulate the secretion of pro-inflammatory cytokines such as interleukin-6 (IL-6) and tumor necrosis factor-alpha (TNF-α) in a controlled manner, which is essential for the activation of the immune response against pathogens. At the same time, they can also promote the production of anti-inflammatory cytokines like interleukin-10 (IL-10) to prevent excessive inflammation [43,44,45]. A recent clinical trial evaluated the effects of a LAB EPS supplement on the immune system of healthy volunteers [46]. The results showed an increase in the number of circulating immune cells and a modulation of cytokine profiles, suggesting a beneficial immunomodulatory effect.

### 5.3. Anti-Tumor

Although the anti-tumor mechanisms of LAB EPSs are still being elucidated, there is growing evidence of their potential in this area. Some LAB EPSs have been shown to inhibit the growth and proliferation of tumor cells. For example, an EPS from a LAB strain was found to induce apoptosis in colon cancer cells in vitro [47,48] The possible mechanisms include the modulation of cell cycle regulators and the activation of apoptotic pathways. Moreover, LAB EPSs may also have an indirect anti-tumor effect by enhancing the host immune response against tumors. They can stimulate the immune cells to recognize and attack tumor cells. While LAB EPSs demonstrate direct tumor-suppressive effects, structural parallels with medicinal plant polysaccharides (e.g., Panax ginseng) suggest common mechanisms involving β-glucan-mediated macrophage activation [49]. These cross-kingdom comparisons highlight the universal therapeutic potential of bioactive polysaccharides [19,37]. However, more research is needed to fully understand the complex interactions between LAB EPSs and tumor cells and to translate these findings into effective cancer therapies.

### 5.4. Anti-Virus

LAB EPSs have exhibited antiviral activities against several viruses. They can interfere with the viral life cycle at different stages. For instance, some EPSs can bind to viral surface proteins, preventing the virus from attaching to host cells. A study on the antiviral effects of LAB EPSs against influenza virus showed that the EPSs could block the binding of the virus to the sialic acid receptors on the surface of host cells [32,50]. High-molecular-weight EPSs (>500 kDa) may sterically hinder membrane fusion processes critical for viral entry, as demonstrated in HIV studies [33]. Additionally, LAB EPSs may also enhance the antiviral immune response of the host. They can activate immune cells such as natural killer (NK) cells, which play a crucial role in the defense against viral infections [51].

### 5.5. Anti-Biofilm

The ability of LAB EPSs to inhibit biofilm formation is another important biological function. Biofilms are communities of microorganisms attached to surfaces and are often associated with persistent infections and food spoilage. LAB EPSs disrupt the formation of biofilms through dual mechanisms: (1) competitive exclusion by adhering to surface receptors (e.g., *E. coli* type-1 fimbriae), and (2) interfering with quorum sensing (QS) signaling molecules (e.g., acyl-homoserine lactones), thereby suppressing virulence gene expression [52,53]. For example, in a study on foodborne pathogens, the addition of LAB EPSs reduced the formation of biofilms by Escherichia coli and Salmonella typhimurium on food contact surfaces [52]. This anti-biofilm property of LAB EPSs has potential applications in food safety and the prevention of medical device-associated infections.

In conclusion, the diverse biological functions of LAB EPSs make them a promising area of research. Their potential applications in health promotion and disease prevention are extensive, but further studies are required to fully understand and harness these benefits.

## 6. Application

### 6.1. Health Benefits

**Figure 5:**
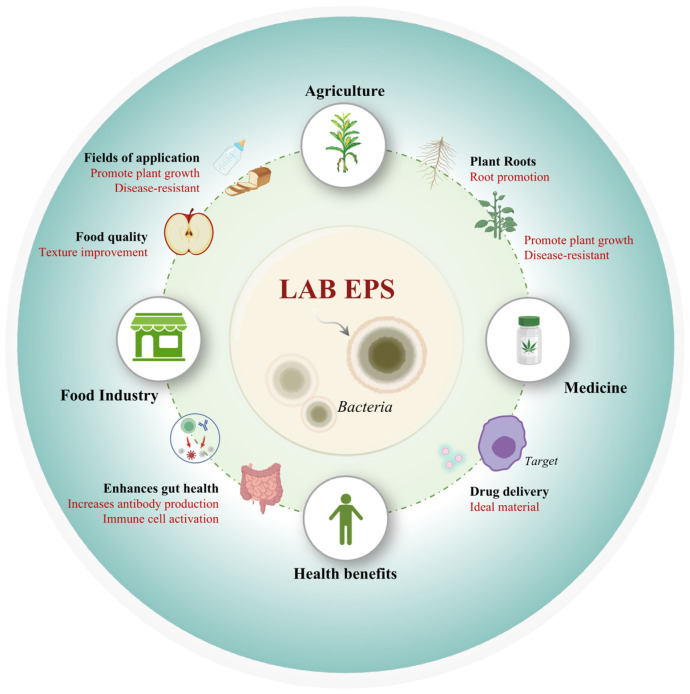
Applications of LAB EPSs in food, pharmaceuticals, and agriculture.

LAB EPSs have shown great potential in promoting human health (Figure 5). They can act as prebiotics, selectively stimulating the growth and activity of beneficial gut microbiota [54]. LAB EPSs lead to an increase in the population of bifidobacteria and lactobacilli in the human gut, which in turn improves gut barrier function and reduces the risk of gastrointestinal disorders [55,56]. Additionally, LAB EPSs possess immunomodulatory properties. Firstly, clinical evidence confirms that LAB EPSs enhance both innate and adaptive immunity: EPSs increased salivary IgA production (+24%) in elderly subjects after 8-week supplementation [57], and they also enhanced NK cell activity (1.7-fold increase) in volunteers receiving EPS-fortified yogurt [58]. Mechanistic studies attribute these effects to EPS-mediated TLR4/NF-κB pathway activation [59]. In a recent trial, patients with recurrent respiratory infections who received LAB EPSs showed a significant reduction in the frequency and severity of infections, accompanied by an increase in the production of specific antibodies and activation of immune cells [56].

### 6.2. Food Industry

In the food industry, LAB EPSs are widely used as natural additives. They can improve the texture and rheological properties of various food products. In dairy products like yogurt and cheese, EPS-producing LAB strains are used to enhance thickness, creaminess, and stability. For instance, the addition of EPSs from a specific LAB strain to yogurt increased its viscosity [57] and prevented syneresis, resulting in a more desirable product texture and longer shelf life [57,58,59]. In bakery products, LAB EPSs can improve dough handling and the volume and softness of the final baked goods [60,61]. Moreover, LAB EPSs can also function as a preservative by inhibiting the growth of spoilage and pathogenic microorganisms in food [53]. Their ability to form a protective barrier around food particles or in the food matrix helps to maintain food quality and safety.

### 6.3. Drug Delivery Systems

The unique properties of LAB EPSs, such as biocompatibility, biodegradability, and the ability to form nanoparticles or microparticles, make them suitable candidates for drug delivery systems [62,63]. EPS-phosphorylated heteropolysaccharides from *Weissella confusa,* have the capacity of forming stable nanoparticles for drug encapsulation due to their biocompatibility and tunable surface charge [64]. The EPS nanoparticles protected the drug from degradation in the gastrointestinal tract and enabled its targeted release at the tumor site, enhancing the therapeutic efficacy and reducing side effects [65]. LAB EPSs can also be modified chemically to attach specific ligands or antibodies, allowing for active targeting of drugs to specific cells or tissues, which holds great promise for personalized medicine.

### 6.4. Agricultural Applications

In agriculture, LAB EPSs have potential applications in plant growth promotion and disease resistance [66,67,68]. Some studies have shown that the application of LAB EPSs to the soil can enhance plant root development. The EPSs can form a matrix around the root, improving soil structure and water retention and facilitating nutrient uptake by the plants. For example, in a greenhouse experiment, plants treated with LAB EPSs had longer and more branched roots compared to the control group. Additionally, LAB EPSs can induce systemic resistance in plants against pathogens. They can activate the plant’s defense mechanisms, such as the production of pathogenesis-related proteins and phytoalexins, thereby reducing the incidence and severity of plant diseases.

In summary, LAB EPSs have a wide range of applications in various fields, from improving human health and food quality to enabling advanced drug delivery and enhancing agricultural productivity. Their versatility and beneficial properties continue to drive research and innovation in multiple industries.

## 7. Conclusions and Perspectives

The study of lactic acid bacteria (LAB) exopolysaccharides (EPSs) has unveiled their structural diversity, functional versatility, and transformative potential across food, health, and agricultural fields. This review synthesizes key advances while delineating critical challenges and future research imperatives.

Key advances include structural and functional insights, biosynthetic innovation, and multifaceted applications. Systematic classification of LAB EPSs based on monosaccharide composition (e.g., homopolysaccharides vs. heteropolysaccharides) and substituent groups (e.g., acetyl, phosphate) has established robust structure–function relationships [18]. These insights underpin their applications in enhancing food texture (e.g., yogurt viscosity [57]) and modulating biological activities (e.g., antioxidant and immunomodulatory effects [34,43]). Advances in genetic and omics tools have unraveled the roles of epsoperons and environmental factors (e.g., Ca^2+^) in EPS production, enabling strain engineering for tailored molecular architectures [22]. Fermentation optimization studies further highlight carbon source selection (e.g., glycerol [31]) and pH control as pivotal for scalable yields [22]. LAB EPSs demonstrate dual utility as natural food additives and bioactive agents, with emerging roles in drug delivery (e.g., nanoparticle encapsulation [64]) and sustainable agriculture (e.g., plant growth promotion [66]).

Despite progress, some barriers hinder translational success, like ambiguous mechanisms, clinical translation, scalability, and sustainability. While LAB EPSs exhibit anti-tumor and antiviral activities [47,50], the molecular interplay between EPS structures (e.g., glycosidic linkages) and cellular targets (e.g., apoptosis pathways) remains poorly resolved. Advanced techniques such as cryo-electron microscopy or metabolomics could bridge this gap [23]. Current evidence for immune modulation is derived predominantly from preclinical models [39,46]. Rigorous human trials are imperative to validate efficacy in pathologies like inflammatory bowel disease or metabolic syndrome [24]. Conventional fermentation methods face economic constraints due to high substrate costs and energy-intensive purification [22]. Innovations in synthetic biology (e.g., minimal genome chassis) or enzymatic assembly in vitro may offer greener alternatives [26].

To fully harness LAB EPS potential, interdisciplinary efforts must be considered: firstly, mechanistic decoding employing single-cell transcriptomics and CRISPR–Cas9 screens to dissect EPS–host interactions at molecular resolution [23]; secondly, clinical validation by designing randomized controlled trials to evaluate EPS efficacy in disease cohorts, leveraging biomarkers (e.g., cytokine profiles) for dose optimization [24]; thirdly, sustainable production, integrating circular economy principles (e.g., agro-industrial waste valorization) with synthetic biology tools to achieve cost-effective, large-scale EPS synthesis [26]; and finally, cross-disciplinary synergy, merging material science with computational modeling (e.g., machine learning-driven EPS design) to engineer next-generation biomaterials for food, medicine, and agriculture [65,69].

LAB EPSs stand at the nexus of tradition and innovation. By addressing these challenges through collaborative research, they may emerge as cornerstones of 21st-century biotechnology, aligning with global sustainability goals [70]. Continued exploration and innovation in this field will undoubtedly lead to new discoveries and the development of novel products and therapies that can improve human and environmental health while also presenting lucrative market opportunities.

## Figures and Tables

**Table 1:** Lactic acid bacteria exopolysaccharide types.

Classification Type	Specific Features	Examples
Structural	Linear or branched sugar chains	Dextran (linear) [10]; EPSs from *Lactobacillus plantarum* (branched) [11]
Type classification	Homopolysaccharide (single sugar)	Fructan [12], polyglactan [13]
	Heteropolysaccharide (multiple sugars)	EPSs produced by *Lactobacillus casei* (glucose, galactose) [14]
Substituent	Acetyl or phosphate substitution	Acetylated EPSs from *S. thermophilus* [15]; Phhosphorylated EPSs from *L. plantarum* [16]

**Table 2:** Lactic acid bacteria exopolysaccharide characterization techniques and information.

Technique	Detection Principle	EPS Information Obtained	Advantages	Limitations	Reference
Fourier Transform Infrared Spectroscopy (FTIR)	1. Measures absorption of infrared radiation by chemical bonds in EPS. 2. Specific vibrational frequencies correspond to functional groups (e.g., hydroxyl, carbonyl).	1. Functional groups (e.g., -OH, C=O). 2. Types of chemical bonds.	1. Rapid and non-destructive. 2. Minimal sample preparation.	1. Limited structural specificity. 2. Cannot resolve linkage types.	[18,21]
Nuclear Magnetic Resonance (NMR)	1. Detects resonance signals of atomic nuclei (e.g., ^1^H, ^13^C) under a magnetic field. 2. Reveals sugar composition, linkage patterns, and substituent positions.	1. Monosaccharide composition. 2. Glycosidic linkage types (α/β). 3. Substituent groups (e.g., acetyl).	1. High structural resolution. 2. Quantitative analysis.	1. Requires pure samples. 2. Expensive instrumentation.	[17,25]
Size Exclusion Chromatography (SEC)	1. Separates EPS molecules by size as they pass through a porous column. 2. Larger molecules elute faster, while smaller ones are retained longer.	1. Molecular weight distribution of the EPS 2. Polydispersity index	1. Direct measurement of molecular weight. 2. Compatible with aqueous solutions.	1. Sensitive to sample concentration. 2. Requires calibration standards.	[18,22]
Scanning Electron Microscopy (SEM)	1. Scans EPS surface with an electron beam. 2. Secondary electrons are detected to create topographic images.	1. Surface morphology. 2. Aggregation state.	1. High-resolution imaging. 2. Visualizes 3D structure.	1. Requires conductive coating. 2. Destructive sample preparation.	[22,23]
Atomic Force Microscopy (AFM)	Measures forces between a sharp tip and EPS surface to generate nanoscale topographic maps.	1. Surface roughness 2. Mechanical properties (e.g., stiffness).	1. Atomic-level resolution. 2. Works in liquid environments.	1. Slow scanning speed. 2. Tip artifacts may occur.	[24,26]

## Data Availability

No new data were created or analyzed in this study. Data sharing is not applicable to this article.

## References

[1] Li Y., Lu X., Chen X., Jiang M., Dong M. (2006). Lactic Acid Bacteria as Functional Starter Cultures for the Food Fermentation Industry. China Dairy Ind..

[2] Korcz E., Varga L. (2021). Exopolysaccharides from Lactic Acid Bacteria: Techno-Functional Application in the Food Industry. Trends Food Sci. Technol..

[3] Gibson G.R., Roberfroid M.B. (1995). Dietary Modulation of the Human Colonic Microbiota: Introducing the Concept of Prebiotics. J. Nutr..

[4] Yan F., Polk D.B. (2002). Probiotic Bacterium Prevents Cytokine-Induced Apoptosis in Intestinal Epithelial Cells. J. Biol. Chem..

[5] Rahbar Saadat Y., Yari Khosroushahi A., Pourghassem Gargari B. (2019). A Comprehensive Review of Anticancer, Immunomodulatory and Health Beneficial Effects of the Lactic Acid Bacteria Exopolysaccharides. Carbohydr. Polym..

[6] Tsai Y.-T., Cheng P.-C., Pan T.M. (2012). The Immunomodulatory Effects of Lactic Acid Bacteria for Improving Immune Functions and Benefits. Appl. Microbiol. Biotechnol..

[7] Tabibloghmany F.S., Ehsandoost E. (2014). An Overview of Healthy and Functionality of Exopolysaccharides Produced by Lactic Acid Bacteria in the Dairy Industry. Eur. J. Nutr. Food Saf..

[8] Tabernero A., González-Garcinuño A., Galán M.A., del Valle E.-M. (2019). Microbial Exopolisaccharides for Biomedical Applications. Materials for Biomedical Engineering.

[9] Xu H., Li Y., Song J., Zhou L., Wu K., Lu X., Zhai X., Wan Z., Gao J. (2024). Highly Active Probiotic Hydrogels Matrixed on Bacterial EPS Accelerate Wound Healing via Maintaining Stable Skin Microbiota and Reducing Inflammation. Bioact. Mater..

[10] Lamothe G., Jolly L., Mollet B., Stingele F. (2002). Genetic and Biochemical Characterization of Exopolysaccharide Biosynthesis by *Lactobacillus delbrueckii* Subsp.. Bulgaricus. Arch. Microbiol..

[11] Notararigo S., Nácher-Vázquez M., Ibarburu I., Werning M.L., de Palencia P.F., Dueñas M.T., Aznar R., López P., Prieto A. (2013). Comparative Analysis of Production and Purification of Homo-and Hetero-Polysaccharides Produced by Lactic Acid Bacteria. Carbohydr. Polym..

[12] Surayot U., Wang J., Seesuriyachan P., Kuntiya A., Tabarsa M., Lee Y., Kim J.-K., Park W., You S. (2014). Exopolysaccharides from Lactic Acid Bacteria: Structural Analysis, Molecular Weight Effect on Immunomodulation. Int. J. Biol. Macromol..

[13] Sanalibaba P., Cakmak G.A. (2016). Exopolysaccharides Production by Lactic Acid Bacteria. Appl. Microbiol. Open Access.

[14] Fuso A., Bancalari E., Castellone V., Caligiani A., Gatti M., Bottari B. (2023). Feeding Lactic Acid Bacteria with Different Sugars: Effect on Exopolysaccharides (EPS) Production and Their Molecular Characteristics. Foods.

[15] Wei Y., Li F., Li L., Huang L., Li Q. (2019). Genetic and Biochemical Characterization of an Exopolysaccharide with in Vitro Antitumoral Activity Produced by *Lactobacillus fermentum* YL-11. Front. Microbiol..

[16] Jiang Y., Zhang M., Zhang Y., Zulewska J., Yang Z. (2021). Calcium (Ca^2+^)-Regulated Exopolysaccharide Biosynthesis in Probiotic *Lactobacillus plantarum* K25 as Analyzed by an Omics Approach. J. Dairy Sci..

[17] Yadav M.K., Song J.H., Vasquez R., Lee J.S., Kim I.H., Kang D.-K. (2024). Methods for Detection, Extraction, Purification, and Characterization of Exopolysaccharides of Lactic Acid Bacteria—A Systematic Review. Foods.

[18] Bajpai V.K., Majumder R., Rather I.A., Kim K. (2016). Extraction, Isolation and Purification of Exopolysaccharide from Lactic Acid Bacteria Using Ethanol Precipitation Method. Bangladesh J. Pharmacol..

[19] Sørensen H.M., Rochfort K.D., Maye S., MacLeod G., Brabazon D., Loscher C., Freeland B. (2022). Exopolysaccharides of Lactic Acid Bacteria: Production, Purification and Health Benefits towards Functional Food. Nutrients.

[20] Raj S.T., Puspanadan S., Gan C.Y., Tan J.S. (2024). Purification of Exopolysaccharide Produced from *Lactobacillus* spp. Using Ionic-Liquid as Adjuvant in Alcohol/Salt-Based Aqueous Two-Phase System for Its Antidiabetic Properties. Int. J. Biol. Macromol..

[21] Wei D.I., Zhang Y., Hua-Xi Y.I., Xue H.A.N., Shu-Mei W., Zhang L. (2018). Research Methods for Structural Analysis of Lactic Acid Bacteria Induced Exopolysaccharides. Chin. J. Anal. Chem..

[22] Jyoti K., Soni K., Chandra R. (2024). Optimization of the Production of Exopolysaccharide (EPS) from Biofilm-Forming Bacterial Consortium Using Different Parameters. Microbe.

[23] Sirin S., Aslim B. (2021). Protective Effect of Exopolysaccharides from Lactic Acid Bacteria against Amyloid Beta1-42induced Oxidative Stress in SH-SY5Y Cells: Involvement of the AKT, MAPK, and NF-ΚB Signaling Pathway. Process Biochem..

[24] Khalil M.A., Sonbol F.I., Al-Madboly L.A., Aboshady T.A., Alqurashi A.S., Ali S.S. (2022). Exploring the Therapeutic Potentials of Exopolysaccharides Derived from Lactic Acid Bacteria and Bifidobacteria: Antioxidant, Antitumor, and Periodontal Regeneration. Front. Microbiol..

[25] Soumya M.P., Nampoothiri K.M. (2021). An Overview of Functional Genomics and Relevance of Glycosyltransferases in Exopolysaccharide Production by Lactic Acid Bacteria. Int. J. Biol. Macromol..

[26] Gudmundsdottir A.B., Brynjolfsdottir A., Olafsdottir E.S., Hardardottir I., Freysdottir J. (2019). Exopolysaccharides from *Cyanobacterium aponinum* Induce a Regulatory Dendritic Cell Phenotype and Inhibit SYK and CLEC7A Expression in Dendritic Cells, T Cells and Keratinocytes. Int. Immunopharmacol..

[27] Xiao L., Xu D., Tang N., Rui X., Zhang Q., Chen X., Dong M., Li W. (2021). Biosynthesis of Exopolysaccharide and Structural Characterization by *Lacticaseibacillus paracasei* ZY-1 Isolated from Tibetan Kefir. Food Chem. Mol. Sci..

[28] Das S. (2022). Genetic Regulation, Biosynthesis and Applications of Extracellular Polysaccharides of the Biofilm Matrix of Bacteria. Carbohydr. Polym..

[29] Gamar-Nourani L., Blondeau K., Simonet J.-M. (1998). Influence of Culture Conditions on Exopolysaccharide Production by *Lactobacillus rhamnosus* Strain C83. J. Appl. Microbiol..

[30] Zhang Y., Dai X., Jin H., Man C., Jiang Y. (2021). The Effect of Optimized Carbon Source on the Synthesis and Composition of Exopolysaccharides Produced by *Lactobacillus paracasei*. J. Dairy Sci..

[31] Minari G.D., Piazza R.D., Sass D.C., Contiero J. (2024). EPS Production by *Lacticaseibacillus casei* Using Glycerol, Glucose, and Molasses as Carbon Sources. Microorganisms.

[32] Bengoa A.A., Llamas M.G., Iraporda C., Dueñas M.T., Abraham A.G., Garrote G.L. (2018). Impact of Growth Temperature on Exopolysaccharide Production and Probiotic Properties of *Lactobacillus paracasei* Strains Isolated from Kefir Grains. Food Microbiol..

[33] Gorret N., Maubois J.L., Engasser J.M., Ghoul M. (2001). Study of the Effects of Temperature, PH and Yeast Extract on Growth and Exopolysaccharides Production by *Propionibacterium acidi-propionici* on Milk Microfiltrate Using a Response Surface Methodology. J. Appl. Microbiol..

[34] Faraki A., Rahmani F. (2021). The Antioxidant Activity of Lactic Acid Bacteria and Probiotics: A Review. J. Food Saf. Hyg..

[35] Kumari M., Dasriya V.L., Ali S.A., Behare P.V. (2024). Evaluation of Antioxidant and Anti-Inflammatory Properties of *Lacticaseibacillus rhamnosus* Ram12-Derived Exopolysaccharide in a D-Galactose-Induced Liver Injury Mouse Model. Int. J. Biol. Macromol..

[36] Aliouche N., Sifour M., Kebsa W., Ouled-Haddar H. (2024). Exploring the Hepatoprotective Potential of the Probiotic *Lactiplantibacillus plantarum* E1K2R2 and Its Exopolysaccharide-Postbiotic on Ibuprofen-Induced Acute Liver Injury in Rats. Naunyn-Schmiedeberg’s Archives of Pharmacology.

[37] Ji X., Guo J., Cao T., Zhang T., Liu Y., Yan Y. (2023). Review on mechanisms and structure-activity relationship of hypoglycemic effects of polysaccharides from natural resources. Food Sci. Hum. Wellness.

[38] Ji X., Peng B., Ding H., Cui B., Nie H., Yan Y. (2023). Purification, structure and biological activity of pumpkin polysaccharides: A review. Food Rev. Int..

[39] Yue Y., Han J., Shen X., Zhu F., Liu Y., Zhang W., Xia W., Wu M. (2024). Structural Characteristics, Immune-Activating Mechanisms in Vitro, and Immunomodulatory Effects in Vivo of the Exopolysaccharide EPS53 from *Streptococcus thermophilus* XJ53. Carbohydr. Polym..

[40] Dicks L.M.T., Grobbelaar M.J. (2021). Double-Barrel Shotgun: Probiotic Lactic Acid Bacteria with Antiviral Properties Modified to Serve as Vaccines. Microorganisms.

[41] Adebayo-Tayo B., Fashogbon R. (2020). In Vitro Antioxidant, Antibacterial, in Vivo Immunomodulatory, Antitumor and Hematological Potential of Exopolysaccharide Produced by Wild Type and Mutant *Lactobacillus delbureckii* Subsp.. Bulgaricus. Heliyon.

[42] Bhawal S., Kumari A., Kapila S., Kapila R. (2022). Biofunctional Attributes of Surface Layer Protein and Cell-Bound Exopolysaccharide from Probiotic *Limosilactobacillus fermentum* (MTCC 5898). Probiotics Antimicrob. Proteins.

[43] Taverniti V., Guglielmetti S. (2011). The Immunomodulatory Properties of Probiotic Microorganisms beyond Their Viability (Ghost Probiotics: Proposal of Paraprobiotic Concept). Genes Nutr..

[44] Bourebaba Y., Marycz K., Mularczyk M., Bourebaba L. (2022). Postbiotics as Potential New Therapeutic Agents for Metabolic Disorders Management. Biomed. Pharmacother..

[45] Xu X., Qiao Y., Peng Q., Shi B., Dia V.P. (2022). Antioxidant and Immunomodulatory Properties of Partially Purified Exopolysaccharide from *Lactobacillus casei* Isolated from Chinese Northeast Sauerkraut. Immunol. Investig..

[46] Jin H., Park J., Li R., Ji G.E., Johnston T.V., Choe D., Park S.-H., Park M.S., Ku S. (2023). A Randomized, Double-Blind, Controlled Human Study: The Efficacy of Exopolysaccharides in Milk Fermented by *Weissella confusa* VP30 (VP30-EPS) to Ameliorate Functional Constipation. J. Funct. Foods.

[47] Deepak V., Ramachandran S., Balahmar R.M., Pandian S.R.K., Sivasubramaniam S.D., Nellaiah H., Sundar K. (2016). In Vitro Evaluation of Anticancer Properties of Exopolysaccharides from *Lactobacillus acidophilus* in Colon Cancer Cell Lines. In Vitro Cell. Dev. Biol.-Anim..

[48] Tukenmez U., Aktas B., Aslim B., Yavuz S. (2019). The Relationship between the Structural Characteristics of Lactobacilli-EPS and Its Ability to Induce Apoptosis in Colon Cancer Cells in Vitro. Sci. Rep..

[49] Ji X., Hou C., Shi M., Yan Y., Liu Y. (2022). An insight into the research concerning *Panax ginseng* C. A. Meyer polysaccharides: A review. Food Rev. Int..

[50] Nagai T., Makino S., Ikegami S., Itoh H., Yamada H. (2011). Effects of Oral Administration of Yogurt Fermented with *Lactobacillus delbrueckii* ssp. Bulgaricus OLL1073R-1 and Its Exopolysaccharides against Influenza Virus Infection in Mice. Int. Immunopharmacol..

[51] Makino S., Sato A., Goto A., Nakamura M., Ogawa M., Chiba Y., Hemmi J., Kano H., Takeda K., Okumura K. (2016). Enhanced Natural Killer Cell Activation by Exopolysaccharides Derived from Yogurt Fermented with *Lactobacillus delbrueckii* ssp. Bulgaricus OLL1073R-1. J. Dairy Sci..

[52] Gómez N.C., Ramiro J.M.P., Quecan B.X.V., de Melo Franco B.D.G. (2016). Use of Potential Probiotic Lactic Acid Bacteria (LAB) Biofilms for the Control of *Listeria monocytogenes, Salmonella* Typhimurium, and *Escherichia coli* O157: H7 Biofilms Formation. Front. Microbiol..

[53] Kavitake D., Tiwari S., Shah I.A., Devi P.B., Delattre C., Reddy G.B., Shetty P.H. (2023). Antipathogenic Potentials of Exopolysaccharides Produced by Lactic Acid Bacteria and Their Food and Health Applications. Food Control.

[54] Werning M.L., Hernández-Alcántara A.M., Ruiz M.J., Soto L.P., Dueñas M.T., López P., Frizzo L.S. (2022). Biological Functions of Exopolysaccharides from Lactic Acid Bacteria and Their Potential Benefits for Humans and Farmed Animals. Foods.

[55] Salazar N., Gueimonde M., De Los Reyes-Gavilán C.G., Ruas-Madiedo P. (2016). Exopolysaccharides Produced by Lactic Acid Bacteria and Bifidobacteria as Fermentable Substrates by the Intestinal Microbiota. Crit. Rev. Food Sci. Nutr..

[56] Dempsey E., Corr S.C. (2022). *Lactobacillus* spp. for Gastrointestinal Health: Current and Future Perspectives. Front. Immunol..

[57] Akar N.Z. (2022). Exopolysaccharides from Lactic Acid Bacteria: Functional Properties and Effects on Yogurt Texture. Osman. Korkut Ata Üniversitesi Fen Bilim. Enstitüsü Derg..

[58] Yang T., Wu K., Wang F., Liang X., Liu Q., Li G., Li Q. (2014). Effect of Exopolysaccharides from Lactic Acid Bacteria on the Texture and Microstructure of Buffalo Yoghurt. Int. Dairy J..

[59] Han X., Yang Z., Jing X., Yu P., Zhang Y., Yi H., Zhang L. (2016). Improvement of the Texture of Yogurt by Use of Exopolysaccharide Producing Lactic Acid Bacteria. BioMed Res. Int..

[60] Abarquero D., Renes E., Fresno J.M., Tornadijo M.E. (2022). Study of Exopolysaccharides from Lactic Acid Bacteria and Their Industrial Applications: A Review. Int. J. Food Sci. Technol..

[61] Milanović V., Osimani A., Garofalo C., Belleggia L., Maoloni A., Cardinali F., Mozzon M., Foligni R., Aquilanti L., Clementi F. (2020). Selection of Cereal-Sourced Lactic Acid Bacteria as Candidate Starters for the Baking Industry. PLoS ONE.

[62] Laubach J., Joseph M., Brenza T., Gadhamshetty V., Sani R.K. (2021). Exopolysaccharide and Biopolymer-Derived Films as Tools for Transdermal Drug Delivery. J. Control. Release.

[63] Adegbolagun T.I., Odeniyi O.A., Odeniyi M.A. (2023). Drug Delivery Applications and Future Prospects of Microbial Exopolysaccharides. Polym. Med..

[64] Amer M., Eldiwany A., Elgammal E., Atwa N.A., Dawoud I., Rashad F.M. (2021). Nano-Exopolysaccharide from the Probiotic Weissella Paramesenteroides MN2C2: Production, Characterization and Anticancer Activity. Egypt. J. Chem..

[65] Raveendran S., Poulose A.C., Yoshida Y., Maekawa T., Kumar D.S. (2013). Bacterial Exopolysaccharide Based Nanoparticles for Sustained Drug Delivery, Cancer Chemotherapy and Bioimaging. Carbohydr. Polym..

[66] Garmasheva I., Tomila T., Kharkhota M., Oleschenko L. (2024). Exopolysaccharides of Lactic Acid Bacteria as Protective Agents against Bacterial and Viral Plant Pathogens. Int. J. Biol. Macromol..

[67] Abhyankar P.S., Gunjal A.B., Kapadnis B.P., Ambade S.V. (2021). Potential of Lactic Acid Bacteria in Plant Growth Promotion. Bhartiya Krishi Anusandhan Patrika.

[68] Raman J., Kim J.-S., Choi K.R., Eun H., Yang D., Ko Y.-J., Kim S.-J. (2022). Application of Lactic Acid Bacteria (LAB) in Sustainable Agriculture: Advantages and Limitations. Int. J. Mol. Sci..

[69] Zannini E., Waters D.M., Coffey A., Arendt E.K. (2016). Production, Properties, and Industrial Food Application of Lactic Acid Bacteria-Derived Exopolysaccharides. Appl. Microbiol. Biotechnol..

[70] Lahmamsi H., Ananou S., Lahlali R., Tahiri A. (2024). Lactic Acid Bacteria as an Eco-Friendly Approach in Plant Production: Current State and Prospects. Folia Microbiol..

